# Breeding ecology of the Asian openbill in eastern Nepal: Larger trees support higher fledgling success

**DOI:** 10.1002/ece3.11504

**Published:** 2024-06-18

**Authors:** Ganesh Tamang, Hem Bahadur Katuwal, Asmit Subba, Nanda Bahadur Singh

**Affiliations:** ^1^ Central Department of Zoology, Institute of Science and Technology Tribhuvan University Kathmandu Nepal; ^2^ Central Campus of Technology, Institute of Science and Technology Tribhuvan University Dharan Nepal; ^3^ Nepal Zoological Society Kathmandu Nepal; ^4^ Center for Integrative Conservation, Xishuangbanna Tropical Botanical Garden, Chinese Academy of Sciences Mengla Yunnan China; ^5^ Nature Conservation and Study Center Kathmandu Nepal

**Keywords:** agricultural land, *Bombax ceiba*, colony size, human habitation, waterbird, wetland

## Abstract

Colonial nesting waterbirds in agricultural landscapes have historically received limited research attention, especially in South Asia. For example, the Asian openbill (*Anastomus oscitans*) is a colonial species that extensively utilizes agricultural landscapes, yet there is a notable lack of substantial studies despite increasing urbanization across these landscapes. We investigated the factors affecting the breeding ecology of Asian openbills in eastern Nepal. We used a grid‐based approach to locate stork colonies and monitored them throughout the breeding season from May to November for three consecutive years (2020–2022). Altogether, we observed a total of 67 active colonies, comprising 4020 active nests, which successfully fledged a total of 7566 chicks. Additionally, most of the colonies were located within areas of human settlements (40%), followed by community forests (33%) and agricultural land (27%). The Asian openbills primarily preferred large trees, such as *Bombax ceiba* (72%), for nesting. The mean height of nesting trees was approximately 4 m taller, the diameter at breast height was twice as large, and the canopy cover area was three times greater than that of non‐nesting trees. The canopy cover area of trees positively influenced the colony size, while colony size positively influenced the fledgling's success. Our study underscores the significance of large trees in providing sufficient space for accommodating a substantial number of openbill nests and fledglings. These findings have significant implications for conservation efforts to protect large trees along with wetlands and agricultural lands, as crucial measures to ensure the sustainable breeding of this nationally vulnerable species.

## Graphical abstract

We found a large number of breeding colonies of Asian openbill across human‐dominated landscapes in eastern lowland Nepal. The Asian openbill showed a preference for large trees, with Bombax ceiba being the most favored species. Our research provides valuable baseline data for the development of comprehensive conservation plans and policies for these storks in eastern Nepal.

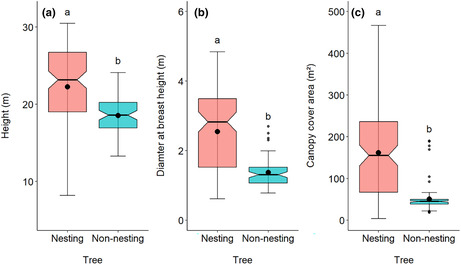

## 1 INTRODUCTION

Waterbirds in South Asia heavily depend on wetlands and agricultural lands throughout the year for foraging and breeding (King et al., 2010; Kittur & Sundar, 2021; Sundar et al., 2016). These habitats provide abundant food, which enhances their breeding performance and productivity (Janiszewski et al., 2014; Sundar, 2006). Moreover, the presence of larger trees in these landscapes, which are maintained by farmers, is essential for various waterbird species (Hilaluddin et al., 2003; Katuwal, Sundar, et al., 2022; Koju et al., 2019). Specifically, these large trees, characterized by their tall height, substantial diameter at breast height (DBH), and expansive canopy cover, facilitate the establishment of larger colony sizes (Clancy & Ford, 2011; Ghimire et al., 2022; Katuwal, Sundar, et al., 2022). This, in turn, significantly impacts the breeding performances of colonial waterbirds, including storks such as the Asian openbills (*Anastomus oscitans*; Mohapatra et al., 2019; Rajković, 2021).

The Asian openbill is a large colonial waterbird with an extensive distribution range in South and Southeast Asia (BirdLife International, 2023). In recent years, it has been gradually expanding its range across most Southeast Asian countries (Lim et al., 2008; Low et al., 2013; Zhang et al., 2022). However, in the Indian subcontinent, this species can be found in all countries, with a larger distribution and population in India compared to other countries (Mohapatra et al., 2019). Owing to its extensive distribution range, the Asian openbill is classified as Least Concern in the IUCN Red List assessment (BirdLife International, 2023). However, the species faces several anthropogenic and natural threats, including the scarcity of nesting trees, agricultural chemicals, hunting, food shortages, and hailstorms (Bhattarai, 2012; Mandal, 2024; Pramanik et al., 2014; Sundar, 2006; Zhang et al., 2022).

Asian openbill colonies are typically found near human habitation, agricultural land, and water bodies, where their prey is abundant (Abidin et al., 2017; Hara et al., 2018; Meganathan & Jeevanadham, 2019; Sundar, 2006); thus, these are critical habitats for them. It primarily feeds on gastropods, particularly *Filopaludina* spp., *Pila* spp., and crabs, along with large insects found in these habitats (Ali & Ripley, 1987; Kahl, 1971; Tamang et al., 2024). It nests mostly in *Bombax ceiba*, *Acacia nilotica*, *Prosopis juliflora*, *Mangifera* spp., *Albizia saman*, *Elaeis guineensis*, *Leucaena leucocephala* and *Neolamarckia cadamba* trees that have great height and wide‐branched canopies, commonly found near human settlements, and farmlands (Abidin et al., 2017; Bhattarai, 2012; Garg, 2016; Koju et al., 2019; Pramanik et al., 2016) and sometimes in forests (Pramanik et al., 2016).

In Nepal, the Asian openbill is categorized as a vulnerable species (Department of National Parks and Wildlife Conservation, Bird Conservation Nepal, 2022), and is primarily found in lowlands below 300 m (Adhikari et al., 2018; Baral, 2008; Inskipp et al., 2016; Katuwal, Rai, et al., 2022; Sundar et al., 2016). Although classified as a resident species, the Asian openbill exhibits some local movements, arriving during the summer and rainy seasons for breeding, with only a few individuals wintering, especially in eastern Nepal (G. Tamang, personal observation); however, this requires detailed seasonal studies. Until now, very few studies have been conducted on the breeding ecology of the Asian openbill (Bhattarai, 2012; Koju et al., 2019; Sundar et al., 2016, 2019), and little is known about the impacts of tree characteristics and land‐use variables on the breeding success of the species. Although higher breeding success rates have been observed in central Nepal (Sundar et al., 2019), very little information is available from other areas. Furthermore, the population is thought to be declining in Nepal (Inskipp et al., 2016), but no detailed study has been carried out yet. Lastly, there is a significant lack of information about where these storks' nest are in Nepal and what habitats they prefer for breeding, especially in the eastern region. This gap is especially concerning because of the ongoing unplanned urbanization in agricultural‐wetland landscapes (Bhattarai et al., 2023; Rimal et al., 2018), and tree felling is rapidly increasing (Katuwal et al., 2024; Katuwal, Sundar, et al., 2022), which could have a major impact on the breeding success of these storks.

We conducted this study to understand the breeding ecology of the Asian openbill in eastern Nepal. The aims of this study were to: (1) identify the primary nesting habitat of the Asian openbill in eastern Nepal; (2) understand the tree species preferred for nesting; and (3) investigate the factors influencing its breeding ecology in that region. Our study is crucial because we provide detailed information on the breeding status of the Asian openbill in eastern Nepal over three consecutive years. This data can be utilized for habitat management and species conservation efforts.

## 2 MATERIALS AND METHODS

### 2.1 Study area

**FIGURE 1:**
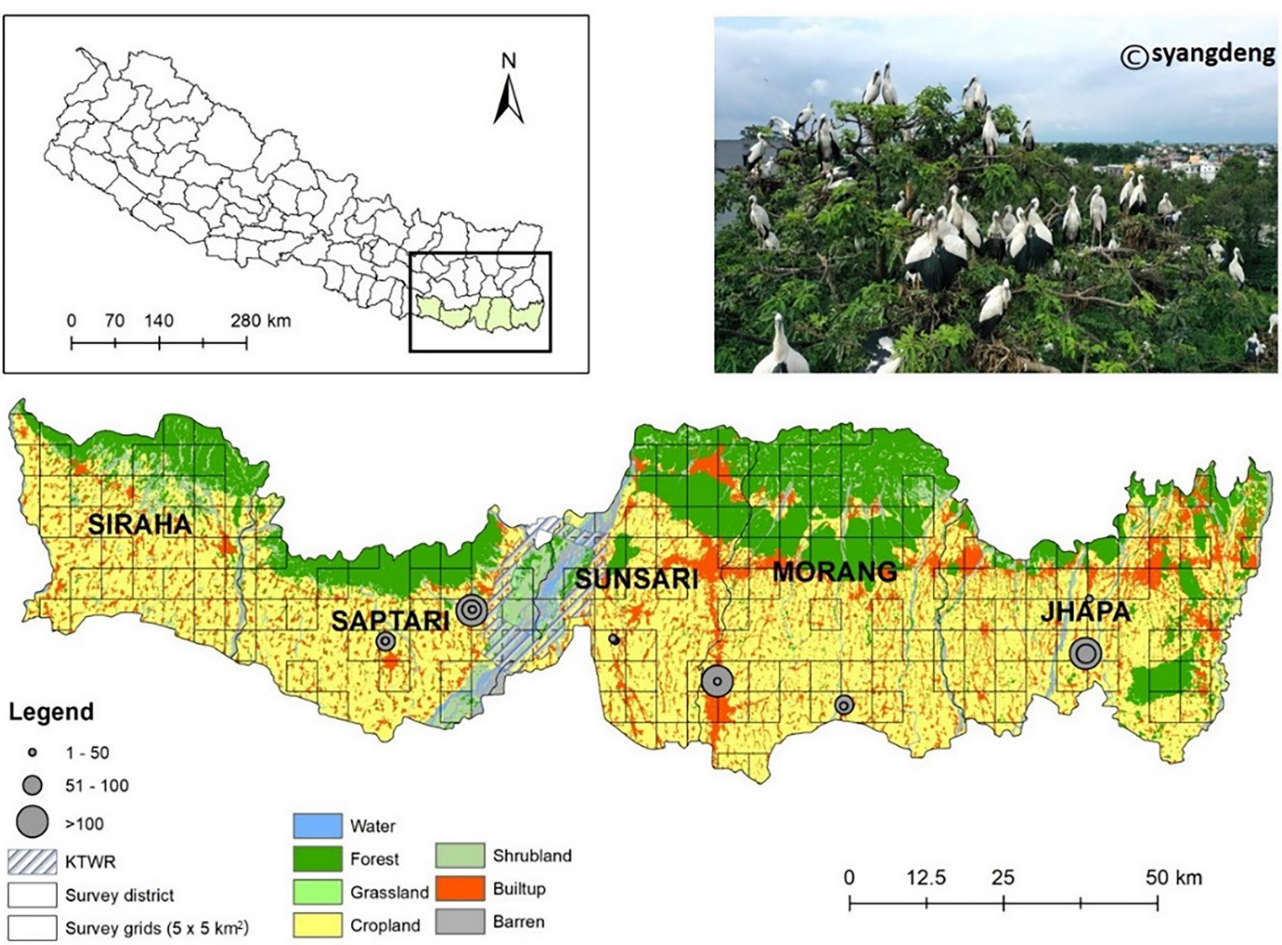
Study area showing the colony locations and colony sizes (number of nests in each colony) of Asian openbill from 2020 to 2022 in eastern Nepal. We did not record any colonies in the Siraha district during the study period.

We conducted this study in various locations within eastern Nepal, primarily in Jhapa, Morang, Sunsari, Saptari, and Siraha districts (Figure 1). The study area is predominantly characterized by agricultural activities, where multiple crops are cultivated throughout the year. Rain and an irrigation network of canals contribute to the water supply for agricultural fields. During the monsoon or rainy season (June–September), flooded rice fields dominate the landscape, while sugarcane, lentils, wheat, and maize are planted in the winter season (November–February). In the warmer summer period, the fields are largely left fallow (March–June, Katuwal, Rai, et al., 2022; Koju et al., 2019). Traditional agriculture practices are followed, with trees planted among crop fields, along roads and canals, and near canals. Wetlands of various sizes, intended for community use, are also present on the landscape and serve human and animal purposes throughout the year.

The climate of lowland Nepal is a mix of tropical and subtropical climates, characterized by hot and humid summers, heavy monsoon rains, and dry winters. From May to November in the years 2020–2022, the monthly mean rainfall in the research locations ranged from 1–874.3 mm, and the lowest and maximum temperatures varied between 13.51°C and 33.96°C. The dominant tree species in the area were *Bombax ceiba*, *Neolamarckia cadamba*, *Eucalyptus* spp., and *Shorea robusta*.

Many forest patches are conserved as community forests or protected reserves, and the extent of forested areas varies among the lowland districts (Ministry of Forests and Environment, 2019; Rimal et al., 2018). Human habitation and some brick factories are steadily increasing in the lowland areas, leading to the shrinking of agricultural land in recent years (Katuwal, Rai, et al., 2022). Densely populated small villages and cities are widespread across all regions. Despite this, it manages to maintain natural elements alongside cultivated lands and artificial features like canals that are intended to facilitate agricultural activities and support bird diversity (Katuwal, Rai, et al., 2022).

### 2.2 Research design

We reviewed the literature and gathered information from locals to identify potential nesting areas. Specifically, we selected five districts: Jhapa, Morang, Sunsari, Saptari, and Siraha (Figure 1). However, we excluded the core area of the Koshi Tappu Wildlife Reserve in Sunsari due to the area's susceptibility to heavy flooding during the rainy season.

We systematically conducted the study and employed a 5 km × 5 km standardized grid approach (Renner et al., 2006), overlaying 353 grids on the study area. However, we excluded 74 grids that did not cover at least 50% of the grid area. From the remaining grids, we randomly subset 140 grids in ArcMap GIS 10.7.1, which covers 50% of the study area and incorporated 7 additional potential grids based on literature and local input, resulting in a total of 147 grids (Figure 1). Moreover, some colonies were discovered outside the designated study grid in subsequent years. As there were only a few colonies, we monitored those colonies as well. Notably, no stork colonies were found in the Siraha district. As a result, our study was conducted in the remaining four districts: Jhapa, Morang, Sunsari, and Saptari (Figure 1).

### 2.3 Nesting colony monitoring

We surveyed the selected area from early May 2020 through the end of November 2022, following the methods previously used in Nepal to study the breeding biology of the Lesser Adjutant and other large waterbirds (Katuwal, Sundar, et al., 2022; Koju et al., 2019; Sundar et al., 2016). In each grid, we traveled all the available motorable routes and enquired with local people to locate stork colonies within the study area (Koju et al., 2019). We conducted our survey in the morning from 6.00 a.m. to 11.00 a.m. and 1.00 p.m. to 5.30 p.m. in the evening. We observed that the majority of Asian openbills began collecting nesting materials in the last week of May. We defined a colony as a nest or a group of nests on trees with contiguous canopies containing at least one chick (Bibby et al., 2000; Sundar et al., 2016), and colony size as the total number of active nests, at least with an egg in them.

To record the position of each colony, we used a Garmin Global Positioning System (GPS 64s). Additionally, we used a field guide to identify the species of nesting trees. The height of the nest tree was measured in meters using a range finder (Bushnell, PRIME 1300), while the DBH was measured in meters using a measuring tape. Additionally, we also measured the tree canopy cover area by measuring the canopy spread across the tree trunk at right angles to one another in four directions. As a result, we calculated the mean canopy cover area as the mean of the four radii. Furthermore, we measured the tree characteristics of the other available trees within a radius of 20 m (considered non‐nesting trees) to understand the differences in tree characteristics between nesting and non‐nesting trees.

Throughout the study, we visited each colony three times a month to record clutch size, chicks, and fledglings until the end of November, when the last group of Asian openbills had completely fledged. The birds do not share nests, but a single female who arrives later may join another pair, indicating a polygamous mating system (Datta & Pal, 1995). To avoid counting nests twice, we marked them near the nest with numbers. We climbed the nesting trees and used a long, foldable stick with a sports camera (GoPro Hero3) attached to it to take photographs for records of the clutch size, number of chicks, and fledglings. In cases where we observed a polygynous nest with more than five eggs in a single clutch, we tagged these with numbers and took photographs using the sports camera. We continued to monitor these nests with the aid of a DJI Mavic 2 Pro Drone until their chicks had successfully fledged. We observed that the young birds were very shy, often leaving their nests and utilizing the nests of other pairs after a month, as seen in painted storks (Suryawanshi & Sundar, 2019). We counted all young birds and adults using aerial photographs taken from a DJI Mavic 2 Pro Drone at a mean height of 22.95 ± 6.85 m. Altogether, we recorded 67 nesting colonies from 2020 to 2022. Specifically, in 2020, we monitored 26 colonies, while in 2021 and 2022, we observed 15 and 26 new colonies, respectively.

### 2.4 Landscape variables

We used a Sentinel image from ESRI to obtain the land use and cover map of the surveyed area for the year 2021 at a 10 × 10 m resolution to explore the effects of land use on the breeding ecology of the species (Karra et al., 2021). We measured the area of agriculture land, wetland (all water bodies), built‐up (human settlements and other areas), and forest (woodland) area within a 5‐km radius of each stork colony. We expected that storks would forage in this 5‐km radius and that the area of agriculture land and wetland may influence both the location of the colony and the storks' nesting success (Sundar et al., 2019). Finally, we measured the distance between each colony and other land uses like human settlements/habitations, wetlands, and agriculture land. These three land use types were chosen since they influence the storks' breeding (Janiszewski et al., 2014; Katuwal et al., 2023; Sundar et al., 2019).

### 2.5 Data analysis

We pooled all data on colony and breeding metrics, including polygamous nests, from 2020 to 2022 to calculate the mean (±SD) and range of colony size, number of chicks, and number of fledglings. We calculated the mean number of chicks fledged per nest by dividing the total number of chicks fledged by the number of nests in each colony. We determined the total population by adding the number of adults to the number of chicks fledged in each colony. We also assessed chick mortality through direct observation and inquiries with local people to ensure we did not miss any instances.

We employed the use versus availability framework (Manly et al., 2002) to understand tree selection by Asian openbills in the adehabitatHS package (Calenge & Basille, 2023) to determine if a particular tree species was selected (used in a higher proportion relative to availability), avoided (used less relative to availability), or used in a similar proportion to availability. We also used the Wilcoxon rank sum test (W) to understand variations in tree characteristics between the nesting and non‐nesting trees.

We performed a multicollinearity test among the predictors, including tree height, DBH, and tree canopy cover area, and the area of farmland, forest, wetland, built‐up, and colony size. We removed variables with a correlation coefficient greater than *r* > .7. For the final analysis, we retained the following variables: tree height, tree canopy cover area, forest area, wetland area, built‐up area, and colony size. Additionally, we performed a spatial autocorrelation analysis using the Moran's I test in the ape package (Paradis & Schliep, 2019). The results showed no statistically significant spatial autocorrelation (*p* > .05).

We used the glmmTMB package (Brooks et al., 2017) to construct a generalized linear mixed model to investigate the factors affecting breeding ecology. We constructed separate models for colony size (total number of active nests in each colony) and fledgling success (total number of chicks fledged from each colony). For colony size, we used tree height, tree canopy cover area, forest area, and wetland area as fixed effects. To understand how tree characteristics, land use, and colony variables affect fledgling success, we added colony size to the existing fixed effects. As we monitored the colonies repeatedly in subsequent years, we considered the year as the random effect in both models. We used negative binomial models to account for the overdispersion raised in the models. We used the ggeffects package (Lüdecke, 2018) to generate predictions for the significant variables of the models. All the analyses were done using the R program, v4.3.2 (R Core Team, 2023).

## 3 RESULTS

### 3.1 Breeding metrics

Altogether, we documented 7566 fledglings from 4020 nests, comprising 11,346 chicks in 67 Asian openbill colonies between 2020 and 2022 in eastern Nepal (Table S1). Among these nests, 100 were polygamous, hosting 441 chicks, of which 318 fledged.

The clutch sizes varied between 1 and 5 for the non‐polygamous nests, whereas they ranged from 6 to 9 for polygamous nests. The mean clutch size across all nests was 3.48 ± 0.36, and the mean chicks fledged per nest during the survey period was 1.89 ± 0.40. Similarly, the mean of eggs per colony was 120.05 ± 98.64, and the mean of chicks was 96.15 ± 78.68, while the fledgling success was 64.11 ± 52.23 for the surveyed period (Table 1). The number of colonies monitored was lowest in 2020, but the number of eggs, chicks, and fledglings' success was higher in that year than in the other years (Table 1). The total population was highest in 2022 (*n* = 6325) while lowest in 2020 (*n* = 4195; Table 1; Table S1).

**TABLE 1:** Details of breeding metrics of Asian openbill in eastern Nepal for 2020–2022.

Year	No. of colony	Colony size (mean ± SD; range)	No. of eggs (mean ± SD; range)	No. of chicks (mean ± SD; range)	Fledgling success (mean ± SD; range)
2020	26	38.07 ± 27.79; 3–130	134.0 ± 101.75; 10–469	108.57 ± 84.82; 6–422	78.30 ± 56.61; 5–278
2021	40	33.10 ± 23.21; 2–96	121.67 ± 87.55; 8–367	100.45 ± 73.72; 7–322	66.67 ± 48.38; 5–207
2022	52	32.80 ± 30.73; 1–123	111.82 ± 106.18; 3–420	86.63 ± 79.58; 3–335	55.05 ± 52.01; 2–222
Total	67	34.06 ± 27.58; 1–130	120.54 ± 98.64; 3–469	96.15 ± 78.68; 3–422	64.11 ± 52.23; 2–278

*Note*: In 2020, we monitored 26 colonies, while in 2021, we observed 15 new colonies, and in 2022, we observed 26 new colonies again, totaling 67 colonies from 2020 to 2022.

The number of colonies was higher in Morang (*n* = 19), followed by Saptari (*n* = 18), and Jhapa (*n* = 17), whereas it was lowest in the Sunsari district (*n* = 13). However, the mean colony size, egg count, chicks, and fledglings were higher in Jhapa while lowest in Sunsari district (Figure S1).

There was 33.32% (*n* = 3780) chick's mortality from 2020 to 2022. Among these years, the lowest mortality was recorded in 2020 (*n* = 787; 27.88%), while the highest mortality was observed in 2022 (*n* = 1642; 36.45%). We identified several potential factors contributing to this mortality, including heavy rainfall causing chicks to fall from nests, extreme heat, insufficient food supplements, choking in early chicks, hunting, and unidentified causes.

### 3.2 Variation in nesting and non‐nesting tree characteristics

Asian openbills utilized a total of five tree species for nesting. The most commonly chosen tree for nesting was *Bombax ceiba*, accounting for 72% (*n* = 85) of the observed nests (Table S2). This was followed by *Neolamarckia cadamba* with 20% (*n* = 24), *Trewia nudiflora* with 5% (*n* = 6), *Albezia* spp. with 2% (*n* = 2), and *Magnifera indica* with 1% (*n* = 1) of the nests. However, the tree selection varied across districts, with *Bombax ceiba* being used more relative to availability and *Neolamarckia cadamba* being used less than available (Table 2). Among the trees not utilized for nesting, *Dalbergia sisso* was the most frequently available species, comprising 32% (*n* = 23) of the observed non‐nesting trees. *Neolamarckia cadamba* accounted for 22% (*n* = 16), while *Eucalyptus* spp. constituted 20% (*n* = 14) of the non‐nesting tree species. *Magnifera indica*, *Tectona grandis*, and *Trewia nudiflora* were also present, albeit in smaller numbers.

**TABLE 2:** Tree species are used by breeding Asian openbill in eastern Nepal.

Species	Jhapa	Morang	Sunsari	Saptari	Combined
*Albezia* spp.			+	0	+
*Bombax ceiba*	+	+	+	0	+
*Magnifera indica*		−	−	0	−
*Neolamarckia cadamba*	−	−	−	0	−
*Trewia nudiflora*	−	−	+		+
*Dalbergia sisso* ^a^	−		−		−
*Eucalyptus* spp.^a^	−	−	−		−
*Tectona grandis* ^a^	−				−

*Note*: Signs indicate trees that were used significantly more relative to availability (+), used in proportion to availability (0), and avoided or used significantly less relative to availability (−). Trees not found in a particular locality do not have a sign. Significance was taken at *p* ≤ .05 level.

^a^ Tree species not used for nesting.

**FIGURE 2:**
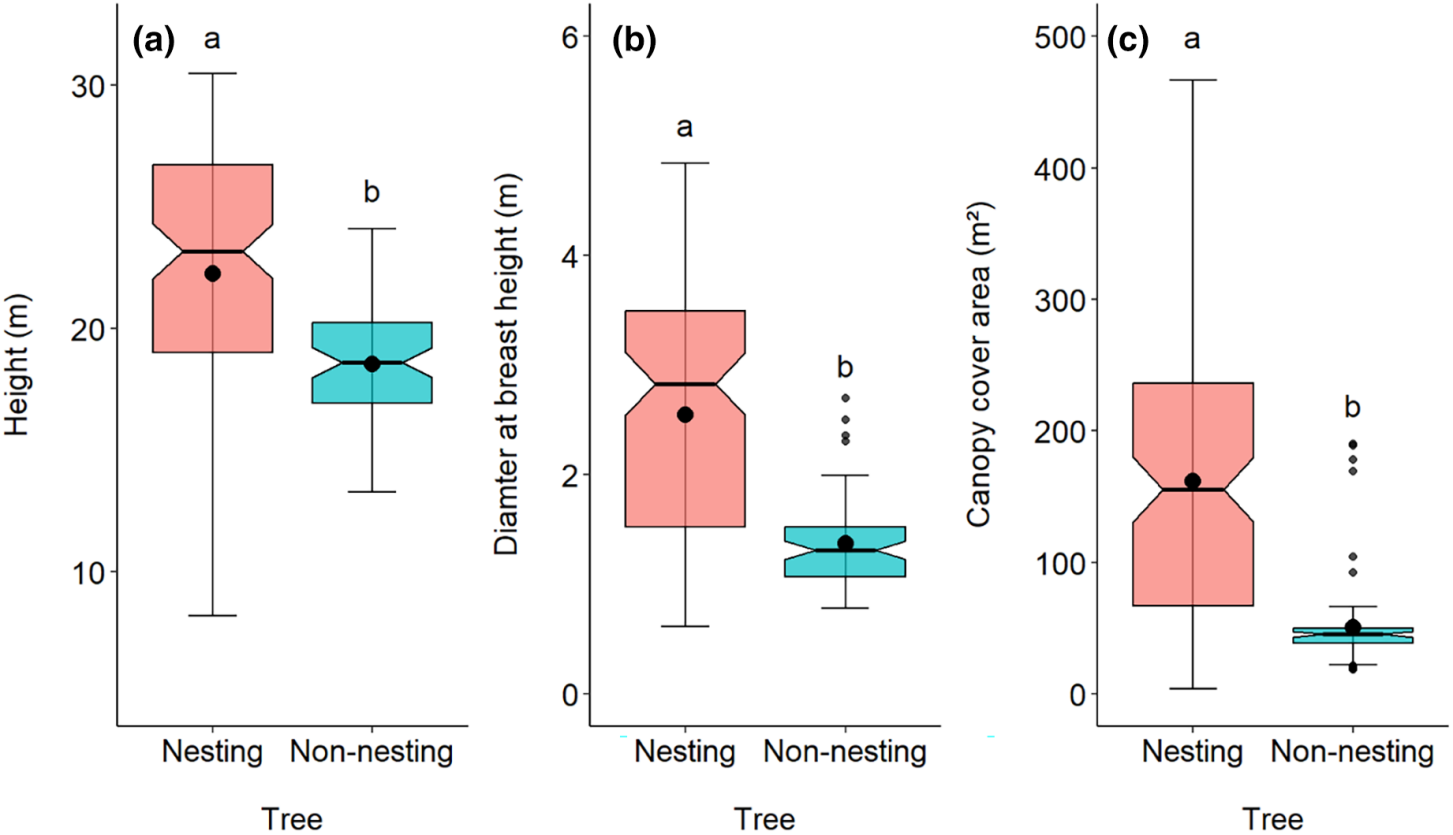
Box plots shows the differences in tree characteristics between non‐nesting and nesting trees used by the Asian openbill in eastern Nepal. The black line in the box is the median, while the black dot is the mean value. The different letters (a, b) above the bar indicate significant differences between each other.

There were significant differences in tree height (*W* = 6149, *p* < .001), DBH (*W* = 6768, *p* < .001), and CCA (*W* = 6622, *p* < .001) between nesting and non‐nesting trees (Figure 2). The mean height of nesting trees (22.25 m ± 5.45) was approximately 4 m taller than non‐nesting trees (18.52 ± 2.62), while the DBH of nesting trees (2.54 m ± 1.10) was twice that of non‐nesting trees (1.37 m ± 0.41), and the CCA of nesting trees (161.75 m^2^ ± 118.11) was three times that of non‐nesting trees (50.29 m^2^ ± 35.42; Figure 2a–c). These three characteristics also varied among the studied districts (Figure S2).

### 3.3 Landscape metrics and factors affecting the fledgling success

The majority of Asian openbill colonies were found within areas of human settlements (40%, *n* = 47), followed by community forest (33%, *n* = 39) and agricultural land (27%, *n* = 32). The mean distance between the colonies and agricultural land was 42.39 ± 47.76 m (range: 2–158 m), while from human settlements, it was 17.38 ± 18.51 m (range: 1–90 m), and from wetlands, it was 76.17 ± 64.76 m (range: 11–220 m).

**FIGURE 3:**
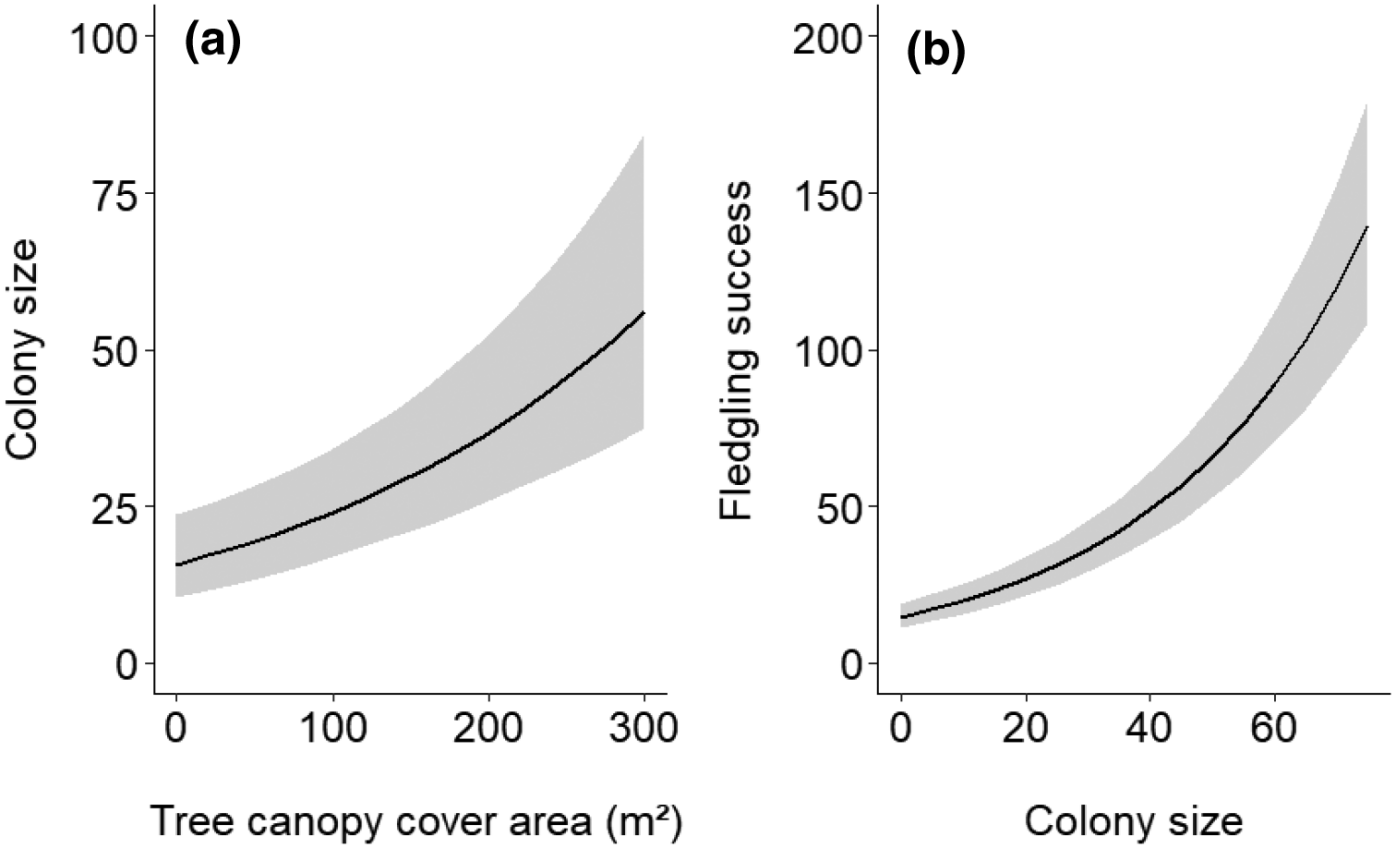
Predicted impact of tree canopy cover area on the colony size (number of nests) (a) and the colony size on the fledgling success (number of chicks fledged) (b) of the Asian openbill in eastern Nepal.

We observed a significant impact only on the tree canopy cover area and colony size in our models (Table 3). The tree canopy cover area positively influenced the colony size, and in turn, colony size positively influenced the fledgling success of the Asian openbill (Figure 3a,b). We did not find any significant impact of the measured land‐use variables on colony size and fledgling success (Table 3).

**TABLE 3:** Factors affecting the colony size and fledgling success of the Asian openbill in eastern Nepal for the year 2020–2022.

Parameter	Estimate	SE	*z*	*p*
*Colony size*				
Intercept	2.77	0.39	6.99	<.001
Tree height	−0.0009	0.01	−0.06	.95
Tree canopy cover area	0.004	0.0007	5.80	<.001
Built‐up area	0.007	0.01	0.77	.43
Wetland area	−0.10	0.11	−0.88	.37
Forest area	−0.04	0.06	−0.71	.47
*Fledgling success*				
Intercept	2.60	0.28	9.05	<.001
Colony size	0.02	0.002	12.59	<.001
Tree height	0.01	0.01	1.52	.12
Tree canopy cover area	0.0002	0.0005	0.40	.68
Built‐up area	0.0009	0.007	0.12	.89
Wetland area	−0.01	0.08	−0.13	.89
Forest area	−0.02	0.05	−0.52	.59

## 4 DISCUSSION

We found several breeding colonies of Asian openbill across various districts of eastern Nepal. These colonies predominantly existed within the areas of human settlements and agricultural lands. Our findings also showed that the presence of large trees and colony size significantly influenced the breeding success of Asian openbill.

The Asian openbill breeds throughout the lowlands of Nepal (Inskipp et al., 2016), although detailed studies on its breeding have been primarily focused on the central lowlands, particularly in the Chitwan, Rupandehi, and Kapilvastu districts (Bhattarai, 2012; Koju et al., 2019; Sundar et al., 2019). Our study from eastern Nepal fills successive gaps and provides more detailed insights studied within Nepal. While the number of fledglings per nest was slightly higher in Central Nepal (Sundar et al., 2019) compared to our study, we monitored a greater number of colonies, colony sizes, fledglings, and population than their study. This increase in colony sizes, fledgling success, and population could be attributed to the extensive three‐year survey we conducted. This suggests that the farmlands and wetlands of eastern Nepal offer suitable breeding habitats for the Asian openbills, akin to other ibises and storks (Karki & Thapa, 2013; Katuwal & Quan, 2022; Katuwal, Rai, et al., 2022). However, we recommend monitoring their non‐breeding populations to understand how this species utilizes these landscapes throughout the year.

We observed polygamy in the Asian openbill, which was also noted by Datta and Pal in 1995. Most stork species are typically monogamous, but the presence of polygamy in the Asian openbill contributes to increased parental care and ultimately enhances breeding success (Datta & Pal, 1995). Polygamous nesting in our study may be attributed to limited nesting space due to the large number of existing nests; however, further investigation is necessary to confirm this and its contribution to breeding success.

The chick mortality observed in our study aligns with findings from Sundar et al. (2019) and other waterbird studies (Gach et al., 2018; Kuiken et al., 1999; Quintana et al., 2022). This mortality may be attributed to various factors such as overcrowding, insufficient food supplementation, choking hazards, extreme temperatures, heavy rainfall or hailstorm, tree felling, and hunting (Bourne et al., 2020; Katuwal, Sundar, et al., 2022; Zhang et al., 2022), including predation by House Crows (*Corvus splendens*) and Rufous Treepies (*Dendrocitta vagabunda*; G. Tamang, personal observation). However, further detailed studies are required to fully understand these factors.

Although *Albezia* spp., and *Trewia nudiflora* were also commonly used trees, the Asian openbill predominantly established their colonies on *Bombax ceiba*, consistent with findings from other studies (Bhattarai, 2012; Mohapatra et al., 2019; Pramanik et al., 2010; Sundar et al., 2016). These trees exhibit greater height and canopy cover, providing suitable nesting conditions for larger colony sizes and minimizing disruption from human activities and storms (Garg, 2016). However, *Neolamarckia cadamba* was used less despite its availability, possibly due to its shorter height and smaller canopies. Nevertheless, the Asian openbill chooses native tree species and tends to avoid the invasive and easily accessible *Eucalyptus* spp. This shows that each stork species has distinct and nuanced preferences for nest trees, as seen in the case of Asian Wooly‐necks, which utilize both local trees and invasive *Eucalyptus* spp. in India (Kittur & Sundar, 2021).

Trees with a greater canopy cover are more likely to support colonial breeders and contribute to the successful breeding and rearing of offspring. Our study showed a significant influence of canopy cover on the colony size of the Asian openbill. Similar findings with breeding success have been reported for Asian openbills (Abidin et al., 2017; Sundar et al., 2016), Lesser Adjutants (Bhattarai, 2012), and Greater Adjutants *Leptoptilus dubios* (Barman & Sharma, 2020; Singha et al., 2002). This shows that the Asian openbills are selective regarding the qualities of their nest trees, such as height and canopy structure, rather than their specific location (Mohapatra et al., 2019; Pramanik et al., 2010). Similarly, colony size also influenced the breeding success of the Asian openbill, as reported in other studies (Lesser Adjutant: Katuwal, Sundar, et al., 2022; Sundar et al., 2019 and Marabou Storks *Leptoptilos crumeniferus*: Monadjem, 2005); Pomeroy, 1977. However, wood storks (*Mycteria americana*), on the other hand, have shown that an increase in the breeding population is associated with a significant rise in the number of colonies rather than an increase in colony size (Ogden et al., 1987). This suggests that studying colony‐level characteristics is essential for understanding the breeding success of colonial waterbirds.

We observed several colonies of the Asian openbill within areas of human habitation and agricultural landscapes, similar to reports for other stork or crane species in South Asia (Katuwal, Sundar, et al., 2022; Kittur & Sundar, 2021; Sharma et al., 2024). This is primarily attributed to the presence of large‐sized nesting trees protected by local communities and possibly the abundance of prey species within agricultural landscapes (Hara et al., 2018; Pramanik et al., 2016; Ratanakorn et al., 2018; Sundar et al., 2019). Additionally, we observed some colonies in forests, indicating that Asian openbills also utilize diverse habitats for breeding. However, we did not observe any significant impact of measured land use variables such as wetland area, built‐up area, and forest area on colony size or the fledgling success of the Asian openbill. This may be due to their greater reliance on agricultural landscapes than other habitats, as the agricultural lands in these areas adhere to traditional practices, are supported by irrigation canals, and provide sufficient food throughout the year (Katuwal, Rai, et al., 2022; Koju et al., 2019; Sundar et al., 2016). Therefore, we recommend protecting agricultural landscapes from haphazard urbanization, reducing the use of agrochemicals, and ensuring water availability throughout the year to optimize habitats for the breeding of the Asian openbill.

## 5 CONCLUSIONS

We observed the large population, number of colonies, and fledgling success of the Asian openbill in eastern Nepal. These birds prefer mostly large trees, like *Bombax ceiba*, in human‐inhabited and agricultural areas for breeding. However, colonies in human‐altered habitats may face threats such as hunting, tree felling, and agrochemical exposure in the future, necessitating continuous monitoring and long‐term studies. Our research offers valuable baseline information for updating the conservation status of the Asian openbill on Nepal's Red List and can inform the development of comprehensive conservation plans and policies for the storks in eastern Nepal.

To enhance the breeding success of the Asian openbill, we suggest prioritizing the protection of large‐sized trees in the region. Furthermore, implementing agricultural policies to regulate urbanization and maintaining irrigation canal networks is vital for enhancing the breeding success of the Asian openbill in eastern Nepal.

## AUTHOR CONTRIBUTIONS

**Ganesh Tamang:** Conceptualization (equal); data curation (equal); formal analysis (equal); funding acquisition (equal); investigation (equal); methodology (equal); project administration (equal); resources (equal); validation (equal); writing – original draft (equal); writing – review and editing (equal). **Hem Bahadur Katuwal:** Conceptualization (equal); data curation (equal); formal analysis (equal); methodology (equal); writing – original draft (equal); writing – review and editing (equal). **Asmit Subba:** Data curation (equal); investigation (equal); writing – review and editing (equal). **Nanda Bahadur Singh:** Conceptualization (equal); methodology (equal); resources (equal); supervision (equal); writing – review and editing (equal).

## CONFLICT OF INTEREST STATEMENT

The authors confirm that they have no known financial interests or personal relationships that could have potentially influenced the work reported in this paper.

## Supporting information


Data S1

## Data Availability

All the relevant data are available in Table S2.
