# Supplementary material for: Breeding ecology of the Asian openbill in eastern Nepal: Larger trees support higher fledgling success
Source: Ecol Evol. 2024 Jun 18;14(6):e11504. doi: 10.1002/ece3.11504 (PMC11183904; doi:10.1002/ece3.11504)
Supplement: Supplementary file 1 — Data S1 [file ECE3-14-e11504-s001.docx]

**Supplementary File**

Table S1: Summary of fledgling success (number of chicks fledged) and population of the Asian Openbill colonies in eastern Nepal for 2020-2022.

| Date | Site | Colony | Colony size | No. of breeding adult | No. of chicks fledged | Total population |
| --- | --- | --- | --- | --- | --- | --- |
| 2020 | Jhapa | RB | 12 | 918 | 865 | 1783 |
| 2020 | Jhapa | TB | 3 | 158 | 124 | 312 |
| 2020 | Morang | DDC | 7 | 434 | 494 | 928 |
| 2020 | Morang | RG | 3 | 351 | 275 | 626 |
| 2020 | Sunsari | INR | 1 | 268 | 278 | 546 |
| 2021 | Jhapa | RB | 13 | 1032 | 1003 | 2035 |
| 2021 | Jhapa | TB | 3 | 145 | 139 | 284 |
| 2021 | Morang | DDC | 8 | 506 | 448 | 954 |
| 2021 | Morang | RG | 5 | 413 | 427 | 840 |
| 2021 | Sunsari | INR | 5 | 306 | 310 | 616 |
| 2021 | Saptari | ML | 6 | 364 | 340 | 704 |
| 2022 | Jhapa | RB | 14 | 1209 | 969 | 2178 |
| 2022 | Jhapa | TB | 2 | 167 | 144 | 311 |
| 2022 | Morang | DDC | 10 | 802 | 642 | 1444 |
| 2022 | Morang | RG | 5 | 249 | 192 | 441 |
| 2022 | Sunsari | INR | 8 | 212 | 141 | 353 |
| 2022 | Saptari | ML | 8 | 352 | 410 | 762 |
| 2022 | Saptari | BR | 5 | 471 | 365 | 836 |

Note: Tulsibari (TB), Raniban (RB), Rangeli (RG), DDC (Dairy Development Corp.), Inaruwa (INR), Bermujhiya (BR) and Maleth (ML)

Table S2: Data of the Asian Openbill in eastern Nepal

| Year | District | Colony  site | Tree | Species | GPS Coordinate | | Colony  size | Polygamous  nest | Ht(m) | DBH(m) | CCA(m^2^) | Egg  count | Chicks | Fledgling success |
| --- | --- | --- | --- | --- | --- | --- | --- | --- | --- | --- | --- | --- | --- | --- |
|  |  |  |  |  | Longitude | Latitude |  |  |  |  |  |  |  |  |
| 2020 | Jhapa | RB1 | Nesting | Bombax ceiba | 87.87798 | 26.52584 | 36 | 0 | 26.94 | 3.2 | 236.91 | 116 | 93 | 84 |
| 2020 | Jhapa | RB1 | Nesting | Bombax ceiba | 87.878 | 26.52563 | 28 | 0 | 25.11 | 4.11 | 171.54 | 95 | 65 | 49 |
| 2020 | Jhapa | RB1 | Nesting | Bombax ceiba | 87.878 | 26.52542 | 96 | 1 | 24 | 4.11 | 466.67 | 272 | 211 | 157 |
| 2020 | Jhapa | RB1 | Nesting | Bombax ceiba | 87.8779 | 26.52536 | 16 | 0 | 23.38 | 3.57 | 137.96 | 46 | 36 | 31 |
| 2020 | Jhapa | RB1 | Nesting | Bombax ceiba | 87.8777 | 26.52526 | 4 | 0 | 27.51 | 3.78 | 81.93 | 15 | 13 | 10 |
| 2020 | Jhapa | RB1 | Nesting | Bombax ceiba | 87.8777 | 26.52539 | 17 | 0 | 24.57 | 3.7 | 432.43 | 51 | 44 | 31 |
| 2020 | Jhapa | RB1 | Nesting | Bombax ceiba | 87.8779 | 26.52654 | 17 | 1 | 28 | 3.5 | 193.5 | 63 | 52 | 38 |
| 2020 | Jhapa | RB1 | Nesting | Bombax ceiba | 87.8793 | 26.52509 | 65 | 4 | 30 | 3.5 | 262.49 | 235 | 196 | 144 |
| 2020 | Jhapa | RB1 | Nesting | Bombax ceiba | 87.8788 | 26.52473 | 56 | 0 | 30 | 3.78 | 393.9 | 163 | 128 | 95 |
| 2020 | Jhapa | RB1 | Nesting | Bombax ceiba | 87.8779 | 26.52479 | 54 | 0 | 30.48 | 3.69 | 230.18 | 160 | 132 | 92 |
| 2020 | Jhapa | RB1 | Nesting | Bombax ceiba | 87.8771 | 26.5246 | 40 | 0 | 30 | 2.74 | 187.1 | 131 | 116 | 79 |
| 2020 | Jhapa | RB1 | Nesting | Bombax ceiba | 87.8788 | 26.5276 | 27 | 1 | 28 | 3.14 | 271.35 | 89 | 76 | 55 |
| 2020 | Jhapa | TB1 | Nesting | Bombax ceiba | 87.8869 | 26.60509 | 52 | 0 | 23.54 | 3.26 | 162.57 | 171 | 153 | 98 |
| 2020 | Jhapa | TB1 | Nesting | Trewia nudiflora | 87.88791 | 26.60475 | 24 | 0 | 17.81 | 1.19 | 81.83 | 39 | 31 | 21 |
| 2020 | Jhapa | TB1 | Nesting | Bombax ceiba | 87.8879 | 26.60474 | 3 | 0 | 15.36 | 1.46 | 65.61 | 10 | 6 | 5 |
| 2020 | Morang | DDC1 | Nesting | Bombax ceiba | 87.2789 | 26.49945 | 51 | 0 | 21.39 | 3.05 | 91.87 | 168 | 139 | 106 |
| 2020 | Morang | DDC1 | Nesting | Bombax ceiba | 87.2791 | 26.49969 | 38 | 1 | 24.6 | 2.92 | 236.91 | 115 | 97 | 80 |
| 2020 | Morang | DDC1 | Nesting | Bombax ceiba | 87.2789 | 26.49988 | 16 | 1 | 16.5 | 2.43 | 43.73 | 57 | 51 | 43 |
| 2020 | Morang | DDC1 | Nesting | Bombax ceiba | 87.2788 | 26.49994 | 9 | 1 | 20.94 | 1.89 | 134.8 | 33 | 29 | 23 |
| 2020 | Morang | DDC1 | Nesting | Bombax ceiba | 87.2785 | 26.49953 | 24 | 1 | 19.69 | 1.86 | 156.87 | 76 | 63 | 55 |
| 2020 | Morang | DDC1 | Nesting | Bombax ceiba | 87.2785 | 26.49947 | 28 | 1 | 21.29 | 3.11 | 150.15 | 93 | 86 | 69 |
| 2020 | Morang | DDC1 | Nesting | Bombax ceiba | 87.2787 | 26.4981 | 48 | 0 | 22 | 2.4 | 61.29 | 157 | 140 | 118 |
| 2020 | Morang | RG1 | Nesting | Bombax ceiba | 87.4846 | 26.45797 | 56 | 1 | 25.3 | 3.26 | 228.64 | 179 | 141 | 101 |
| 2020 | Morang | RG1 | Nesting | Bombax ceiba | 87.4844 | 26.46139 | 72 | 4 | 28.5 | 3.32 | 214.19 | 268 | 154 | 94 |
| 2020 | Morang | RG1 | Nesting | Bombax ceiba | 87.484 | 26.46093 | 43 | 4 | 30 | 4.84 | 262.49 | 213 | 149 | 80 |
| 2020 | Sunsari | INR1 | Nesting | Bombax ceiba | 87.11411 | 26.5629 | 133 | 2 | 24.69 | 2.83 | 232.74 | 469 | 422 | 278 |
| 2021 | Jhapa | RB1 | Nesting | Bombax ceiba | 87.87798 | 26.52584 | 40 | 2 | 26.94 | 3.2 | 236.91 | 146 | 122 | 86 |
| 2021 | Jhapa | RB1 | Nesting | Bombax ceiba | 87.878 | 26.52563 | 21 | 1 | 25.11 | 4.11 | 171.54 | 74 | 61 | 39 |
| 2021 | Jhapa | RB1 | Nesting | Bombax ceiba | 87.878 | 26.52542 | 89 | 2 | 24 | 4.11 | 466.67 | 312 | 250 | 153 |
| 2021 | Jhapa | RB1 | Nesting | Bombax ceiba | 87.8779 | 26.52536 | 6 | 0 | 23.38 | 3.57 | 137.96 | 19 | 14 | 8 |
| 2021 | Jhapa | RB1 | Nesting | Bombax ceiba | 87.8777 | 26.52539 | 25 | 0 | 24.57 | 3.7 | 432.43 | 87 | 71 | 36 |
| 2021 | Jhapa | RB1 | Nesting | Bombax ceiba | 87.8779 | 26.52654 | 30 | 0 | 28 | 3.5 | 193.5 | 104 | 89 | 58 |
| 2021 | Jhapa | RB1 | Nesting | Bombax ceiba | 87.8793 | 26.52509 | 52 | 0 | 30 | 3.5 | 262.49 | 176 | 152 | 106 |
| 2021 | Jhapa | RB1 | Nesting | Bombax ceiba | 87.8788 | 26.52473 | 57 | 0 | 30 | 3.78 | 393.9 | 217 | 188 | 129 |
| 2021 | Jhapa | RB1 | Nesting | Bombax ceiba | 87.8779 | 26.52479 | 51 | 0 | 30.48 | 3.69 | 230.18 | 197 | 170 | 119 |
| 2021 | Jhapa | RB1 | Nesting | Bombax ceiba | 87.8771 | 26.5246 | 43 | 3 | 30 | 2.74 | 187.1 | 153 | 136 | 92 |
| 2021 | Jhapa | RB1 | Nesting | Bombax ceiba | 87.8788 | 26.5276 | 36 | 0 | 28 | 3.14 | 271.35 | 138 | 110 | 72 |
| 2021 | Jhapa | RB2 | Nesting | Bombax ceiba | 87.879349 | 26.52507 | 14 | 2 | 23.16 | 2.83 | 258.1 | 49 | 43 | 28 |
| 2021 | Jhapa | RB2 | Nesting | Bombax ceiba | 87.879324 | 26.52506 | 45 | 2 | 24.07 | 2.83 | 279.95 | 161 | 131 | 77 |
| 2021 | Jhapa | TB1 | Nesting | Bombax ceiba | 87.8869 | 26.60509 | 4 | 0 | 23.54 | 3.26 | 162.57 | 16 | 13 | 10 |
| 2021 | Jhapa | TB1 | Nesting | Trewia nudiflora | 87.88791 | 26.60475 | 46 | 3 | 17.81 | 1.19 | 81.83 | 169 | 140 | 90 |
| 2021 | Jhapa | TB1 | Nesting | Bombax ceiba | 87.8879 | 26.60474 | 21 | 0 | 15.36 | 1.46 | 65.61 | 71 | 60 | 39 |
| 2021 | Morang | DDC1 | Nesting | Bombax ceiba | 87.2789 | 26.49945 | 45 | 2 | 21.39 | 3.05 | 91.87 | 162 | 130 | 87 |
| 2021 | Morang | DDC1 | Nesting | Bombax ceiba | 87.2791 | 26.49969 | 58 | 0 | 24.6 | 2.92 | 236.91 | 208 | 168 | 106 |
| 2021 | Morang | DDC1 | Nesting | Bombax ceiba | 87.2789 | 26.49988 | 26 | 0 | 16.5 | 2.43 | 43.73 | 99 | 75 | 50 |
| 2021 | Morang | DDC1 | Nesting | Bombax ceiba | 87.2788 | 26.49994 | 10 | 1 | 20.94 | 1.89 | 134.8 | 36 | 26 | 22 |
| 2021 | Morang | DDC1 | Nesting | Bombax ceiba | 87.2785 | 26.49953 | 43 | 1 | 19.69 | 1.86 | 156.87 | 141 | 115 | 78 |
| 2021 | Morang | DDC1 | Nesting | Bombax ceiba | 87.2785 | 26.49947 | 20 | 2 | 21.29 | 3.11 | 150.15 | 59 | 45 | 31 |
| 2021 | Morang | DDC2 | Nesting | Bombax ceiba | 87.278823 | 26.49974 | 5 | 0 | 21 | 2.92 | 67.14 | 19 | 15 | 10 |
| 2021 | Morang | DDC2 | Nesting | Neolamarckia cadamba | 87.278631 | 26.49963 | 41 | 2 | 13.6 | 0.68 | 66.47 | 134 | 94 | 64 |
| 2021 | Morang | RG1 | Nesting | Bombax ceiba | 87.4846 | 26.45797 | 45 | 0 | 25.3 | 3.26 | 228.64 | 177 | 148 | 95 |
| 2021 | Morang | RG1 | Nesting | Bombax ceiba | 87.4844 | 26.46139 | 52 | 2 | 28.5 | 3.32 | 214.19 | 191 | 162 | 114 |
| 2021 | Morang | RG1 | Nesting | Bombax ceiba | 87.484 | 26.46093 | 97 | 9 | 30 | 4.84 | 262.49 | 367 | 322 | 207 |
| 2021 | Morang | RG2 | Nesting | Bombax ceiba | 87.484032 | 26.45758 | 2 | 0 | 27.43 | 3.48 | 33.19 | 8 | 7 | 5 |
| 2021 | Morang | RG2 | Nesting | Neolamarckia cadamba | 87.484462 | 26.46121 | 5 | 0 | 14.08 | 1.28 | 6.64 | 8 | 7 | 6 |
| 2021 | Sunsari | INR1 | Nesting | Bombax ceiba | 87.11411 | 26.5629 | 65 | 3 | 24.69 | 2.83 | 232.74 | 260 | 208 | 142 |
| 2021 | Sunsari | INR2 | Nesting | Bombax ceiba | 87.114482 | 26.56327 | 47 | 2 | 18.6 | 1.4 | 13.45 | 174 | 154 | 105 |
| 2021 | Sunsari | INR2 | Nesting | Bombax ceiba | 87.111617 | 26.56342 | 14 | 0 | 11 | 0.64 | 9.63 | 38 | 33 | 22 |
| 2021 | Sunsari | INR2 | Nesting | Bombax ceiba | 87.111435 | 26.56303 | 11 | 1 | 10.8 | 0.87 | 6.44 | 34 | 28 | 19 |
| 2021 | Sunsari | INR2 | Nesting | Neolamarckia cadamba | 87.112236 | 26.5638 | 13 | 0 | 13.6 | 1.36 | 11.84 | 38 | 33 | 22 |
| 2021 | Saptari | ML1 | Nesting | Bombax ceiba | 86.7429 | 26.5683 | 51 | 3 | 21.94 | 2.1 | 81.49 | 203 | 160 | 113 |
| 2021 | Saptari | ML1 | Nesting | Magnifera spp. | 86.7432 | 26.5683 | 42 | 2 | 21.03 | 1.76 | 189.62 | 153 | 108 | 75 |
| 2021 | Saptari | ML1 | Nesting | Neolamarckia cadamba | 86.7433 | 26.5682 | 5 | 0 | 18.28 | 1.55 | 154.07 | 12 | 11 | 8 |
| 2021 | Saptari | ML1 | Nesting | Neolamarckia cadamba | 86.7433 | 26.5685 | 5 | 0 | 19.56 | 0.61 | 204.61 | 11 | 9 | 6 |
| 2021 | Saptari | ML1 | Nesting | Neolamarckia cadamba | 86.740863 | 26.56998 | 19 | 0 | 22 | 1.22 | 165.04 | 74 | 61 | 42 |
| 2021 | Saptari | ML1 | Nesting | Bombax ceiba | 86.740912 | 26.56986 | 57 | 1 | 23.96 | 2.28 | 388.42 | 172 | 149 | 96 |
| 2022 | Jhapa | RB1 | Nesting | Bombax ceiba | 87.87798 | 26.52584 | 35 | 0 | 26.94 | 3.2 | 236.91 | 112 | 94 | 56 |
| 2022 | Jhapa | RB1 | Nesting | Bombax ceiba | 87.878 | 26.52563 | 36 | 0 | 25.11 | 4.11 | 171.54 | 116 | 88 | 50 |
| 2022 | Jhapa | RB1 | Nesting | Bombax ceiba | 87.878 | 26.52542 | 121 | 7 | 24 | 4.11 | 466.67 | 419 | 335 | 222 |
| 2022 | Jhapa | RB1 | Nesting | Bombax ceiba | 87.8779 | 26.52536 | 17 | 0 | 23.38 | 3.57 | 137.96 | 53 | 43 | 30 |
| 2022 | Jhapa | RB1 | Nesting | Bombax ceiba | 87.8777 | 26.52526 | 1 | 0 | 27.51 | 3.78 | 81.93 | 3 | 3 | 2 |
| 2022 | Jhapa | RB1 | Nesting | Bombax ceiba | 87.8777 | 26.52539 | 35 | 0 | 24.57 | 3.7 | 432.43 | 112 | 95 | 64 |
| 2022 | Jhapa | RB1 | Nesting | Bombax ceiba | 87.8779 | 26.52654 | 21 | 0 | 28 | 3.5 | 193.5 | 74 | 59 | 41 |
| 2022 | Jhapa | RB1 | Nesting | Bombax ceiba | 87.8793 | 26.52509 | 52 | 1 | 30 | 3.5 | 262.49 | 161 | 127 | 71 |
| 2022 | Jhapa | RB1 | Nesting | Bombax ceiba | 87.8788 | 26.52473 | 65 | 0 | 30 | 3.78 | 393.9 | 214 | 145 | 82 |
| 2022 | Jhapa | RB1 | Nesting | Bombax ceiba | 87.8779 | 26.52479 | 47 | 0 | 30.48 | 3.69 | 230.18 | 148 | 120 | 72 |
| 2022 | Jhapa | RB1 | Nesting | Bombax ceiba | 87.8771 | 26.5246 | 49 | 1 | 30 | 2.74 | 187.1 | 161 | 134 | 87 |
| 2022 | Jhapa | RB1 | Nesting | Bombax ceiba | 87.8788 | 26.5276 | 42 | 0 | 28 | 3.14 | 271.35 | 136 | 107 | 70 |
| 2022 | Jhapa | RB2 | Nesting | Bombax ceiba | 87.879349 | 26.52507 | 5 | 0 | 23.16 | 2.83 | 258.1 | 13 | 11 | 8 |
| 2022 | Jhapa | RB2 | Nesting | Bombax ceiba | 87.879324 | 26.52506 | 75 | 0 | 24.07 | 2.83 | 279.95 | 229 | 191 | 114 |
| 2022 | Jhapa | TB1 | Nesting | Bombax ceiba | 87.88791 | 26.60475 | 47 | 1 | 14.54 | 1.68 | 86.69 | 114 | 92 | 52 |
| 2022 | Jhapa | TB1 | Nesting | Bombax ceiba | 87.8879 | 26.60474 | 36 | 0 | 15.36 | 1.46 | 65.61 | 161 | 131 | 92 |
| 2022 | Morang | DDC1 | Nesting | Bombax ceiba | 87.2789 | 26.49945 | 7 | 0 | 21.39 | 3.048 | 91.87 | 24 | 18 | 12 |
| 2022 | Morang | DDC1 | Nesting | Bombax ceiba | 87.2791 | 26.49969 | 42 | 0 | 24.6 | 2.92 | 236.91 | 152 | 125 | 68 |
| 2022 | Morang | DDC1 | Nesting | Bombax ceiba | 87.2789 | 26.49988 | 14 | 1 | 16.5 | 2.43 | 43.73 | 54 | 43 | 27 |
| 2022 | Morang | DDC1 | Nesting | Bombax ceiba | 87.2788 | 26.49994 | 14 | 1 | 20.94 | 1.89 | 134.8 | 57 | 47 | 31 |
| 2022 | Morang | DDC1 | Nesting | Bombax ceiba | 87.2785 | 26.49953 | 26 | 0 | 19.69 | 1.86 | 156.87 | 91 | 74 | 44 |
| 2022 | Morang | DDC1 | Nesting | Bombax ceiba | 87.2785 | 26.49947 | 20 | 0 | 21.29 | 3.11 | 150.15 | 73 | 60 | 49 |
| 2022 | Morang | DDC2 | Nesting | Bombax ceiba | 87.278823 | 26.49974 | 17 | 0 | 21 | 2.92 | 67.14 | 50 | 39 | 27 |
| 2022 | Morang | DDC2 | Nesting | Neolamarckia cadamba | 87.278631 | 26.49963 | 115 | 3 | 13.6 | 0.68 | 66.47 | 420 | 302 | 202 |
| 2022 | Morang | DDC3 | Nesting | Neolamarckia cadamba | 87.27905 | 26.49986 | 133 | 1 | 16.9 | 1.34 | 218.5 | 376 | 238 | 162 |
| 2022 | Morang | DDC3 | Nesting | Neolamarckia cadamba | 87.278955 | 26.49955 | 10 | 0 | 8.2 | 1.12 | 11.99 | 38 | 30 | 20 |
| 2022 | Morang | RG1 | Nesting | Bombax ceiba | 87.484 | 26.46093 | 68 | 2 | 30 | 4.84 | 262.49 | 230 | 194 | 116 |
| 2022 | Morang | RG3 | Nesting | Trewia nudiflora | 87.483474 | 26.4587 | 26 | 3 | 20 | 1.19 | 13.45 | 81 | 65 | 45 |
| 2022 | Morang | RG3 | Nesting | Neolamarckia cadamba | 87.48313 | 26.45885 | 9 | 0 | 25 | 1.22 | 9.63 | 26 | 21 | 9 |
| 2022 | Morang | RG3 | Nesting | Trewia nudiflora | 87.483254 | 26.45883 | 9 | 0 | 19 | 0.91 | 11.46 | 24 | 20 | 13 |
| 2022 | Morang | RG3 | Nesting | Bombax ceiba | 87.483229 | 26.45904 | 10 | 0 | 21.33 | 2.62 | 3.91 | 26 | 20 | 9 |
| 2022 | Sunsari | INR3 | Nesting | Neolamarckia cadamba | 87.114861 | 26.56323 | 7 | 0 | 13 | 0.76 | 6.44 | 24 | 18 | 11 |
| 2022 | Sunsari | INR3 | Nesting | Trewia nudiflora | 87.114848 | 26.56329 | 7 | 0 | 15 | 1.28 | 10.52 | 9 | 8 | 5 |
| 2022 | Sunsari | INR3 | Nesting | Bombax ceiba | 87.114609 | 26.56327 | 8 | 0 | 22 | 0.76 | 7.95 | 12 | 9 | 6 |
| 2022 | Sunsari | INR3 | Nesting | Trewia nudiflora | 87.112729 | 26.56368 | 15 | 0 | 25 | 1.37 | 11.46 | 24 | 15 | 10 |
| 2022 | Sunsari | INR3 | Nesting | Neolamarckia cadamba | 87.111018 | 26.56667 | 23 | 2 | 20 | 1.58 | 7.15 | 86 | 69 | 45 |
| 2022 | Sunsari | INR3 | Nesting | Albezia spp. | 87.110931 | 26.56675 | 10 | 0 | 19.6 | 1.64 | 11.46 | 36 | 28 | 15 |
| 2022 | Sunsari | INR3 | Nesting | Neolamarckia cadamba | 87.110279 | 26.56604 | 23 | 0 | 13.7 | 1.64 | 10.52 | 76 | 59 | 34 |
| 2022 | Sunsari | INR3 | Nesting | Neolamarckia cadamba | 87.110323 | 26.56613 | 13 | 0 | 12.2 | 1.1 | 4.62 | 40 | 23 | 15 |
| 2022 | Saptari | ML1 | Nesting | Neolamarckia cadamba | 86.740912 | 26.56986 | 61 | 1 | 22 | 1.22 | 165.04 | 232 | 162 | 96 |
| 2022 | Saptari | ML2 | Nesting | Neolamarckia cadamba | 86.74046 | 26.56995 | 21 | 0 | 15 | 1.58 | 94.98 | 76 | 66 | 43 |
| 2022 | Saptari | ML2 | Nesting | Neolamarckia cadamba | 86.740077 | 26.56945 | 10 | 0 | 19 | 1.19 | 113.04 | 40 | 29 | 18 |
| 2022 | Saptari | ML2 | Nesting | Albezia spp. | 86.739777 | 26.56957 | 27 | 1 | 21 | 1.52 | 50.24 | 92 | 63 | 33 |
| 2022 | Saptari | ML2 | Nesting | Neolamarckia cadamba | 86.739704 | 26.56966 | 32 | 0 | 22 | 1.58 | 94.98 | 141 | 104 | 69 |
| 2022 | Saptari | ML2 | Nesting | Neolamarckia cadamba | 86.739658 | 26.57034 | 15 | 0 | 27 | 1.52 | 103.81 | 56 | 47 | 29 |
| 2022 | Saptari | ML2 | Nesting | Neolamarckia cadamba | 86.740291 | 26.57 | 9 | 0 | 16 | 1.16 | 28.26 | 35 | 26 | 19 |
| 2022 | Saptari | ML2 | Nesting | Neolamarckia cadamba | 86.740405 | 26.57007 | 50 | 1 | 15.26 | 1.83 | 113.04 | 184 | 153 | 103 |
| 2022 | Saptari | BR1 | Nesting | Bombax ceiba | 86.882618 | 26.61169 | 131 | 5 | 26.12 | 4.75 | 314 | 401 | 307 | 203 |
| 2022 | Saptari | BR1 | Nesting | Bombax ceiba | 86.881562 | 26.61302 | 63 | 0 | 24.5 | 3.12 | 226.86 | 188 | 155 | 99 |
| 2022 | Saptari | BR1 | Nesting | Neolamarckia cadamba | 86.882735 | 26.61332 | 6 | 0 | 17.6 | 1.35 | 119.19 | 14 | 12 | 8 |
| 2022 | Saptari | BR1 | Nesting | Neolamarckia cadamba | 86.883246 | 26.61294 | 19 | 0 | 10.4 | 1.61 | 106.89 | 58 | 44 | 32 |
| 2022 | Saptari | BR1 | Nesting | Neolamarckia cadamba | 86.883099 | 26.61286 | 14 | 0 | 11.68 | 1.43 | 78.5 | 43 | 37 | 23 |


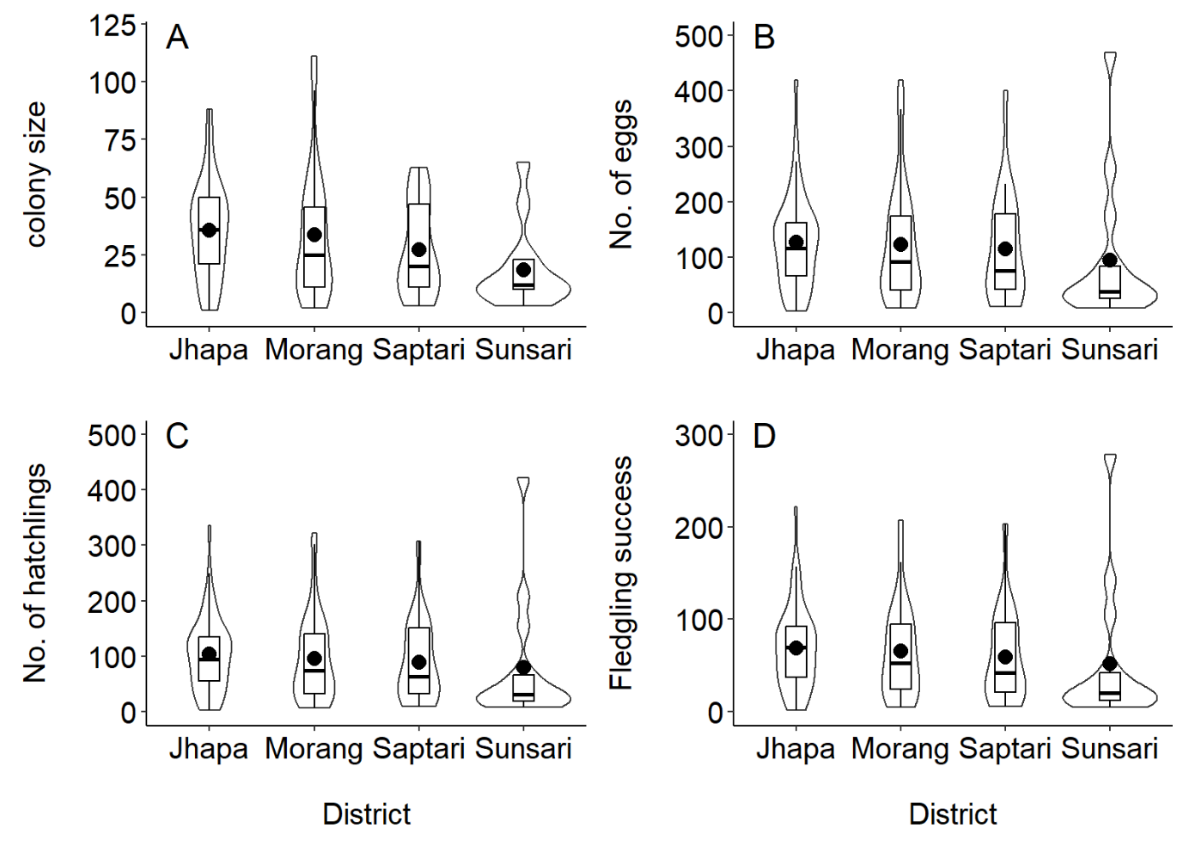


Figure S1: Violin plots showing the breeding metrics of Asian Openbill across study areas for 2020-2022. The black line in the box plot within the violin represents the median, and the dot represents the mean value.


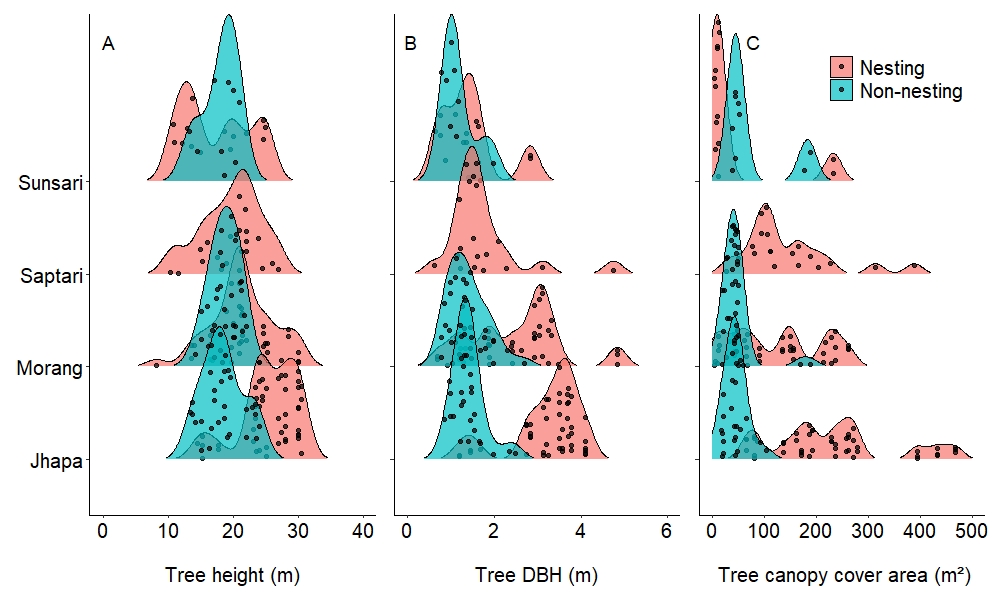


Figure S2: Ridgeline density plots showing the differences in tree characteristics between non-nesting and nesting trees used by the Asian Openbill in eastern Nepal.
